# Longitudinal analyses of CLL in mice identify leukemia-related clonal changes including a *Myc* gain predicting poor outcome in patients

**DOI:** 10.1038/s41375-021-01381-4

**Published:** 2021-08-20

**Authors:** Selcen Öztürk, Yashna Paul, Saira Afzal, Irene Gil-Farina, Anna Jauch, Peter-Martin Bruch, Verena Kalter, Bola Hanna, Lavinia Arseni, Philipp M. Roessner, Manfred Schmidt, Stephan Stilgenbauer, Sascha Dietrich, Peter Lichter, Marc Zapatka, Martina Seiffert

**Affiliations:** 1 Division of Molecular Genetics, German Cancer Research Center (DKFZ), Heidelberg, Germany; 2 Department of Translational Oncology, National Center for Tumor Diseases and German Cancer Research Center (DKFZ), Heidelberg, Germany; 3 GeneWerk GmbH, Heidelberg, Germany; 4 Institute of Human Genetics, University of Heidelberg, Heidelberg, Germany; 5 Department of Medicine V, Hematology, Oncology and Rheumatology, University of Heidelberg, Heidelberg, Germany; 6 Department of Internal Medicine III, Ulm University, Ulm, Germany

**Keywords:** Chronic lymphocytic leukaemia, Cancer models, Cancer genomics

## Abstract

Chronic lymphocytic leukemia (CLL) is a B-cell malignancy mainly occurring at an advanced age with no single major genetic driver. Transgenic expression of *TCL1* in B cells leads after a long latency to a CLL-like disease in aged Eµ-*TCL1* mice suggesting that *TCL1* overexpression is not sufficient for full leukemic transformation. In search for secondary genetic events and to elucidate the clonal evolution of CLL, we performed whole exome and B-cell receptor sequencing of longitudinal leukemia samples of Eµ-*TCL1* mice. We observed a B-cell receptor stereotypy, as described in patients, confirming that CLL is an antigen-driven disease. Deep sequencing showed that leukemia in Eµ-*TCL1* mice is mostly monoclonal. Rare oligoclonality was associated with inability of tumors to develop disease upon adoptive transfer in mice. In addition, we identified clonal changes and a sequential acquisition of mutations with known relevance in CLL, which highlights the genetic similarities and therefore, suitability of the Eµ-*TCL1* mouse model for progressive CLL. Among them, a recurrent gain of chromosome 15, where *Myc* is located, was identified in almost all tumors in Eµ-*TCL1* mice. Interestingly, amplification of 8q24, the chromosomal region containing *MYC* in humans, was associated with worse outcome of patients with CLL.

## Abstract (web summary)

Clonal evolution and genomic alterations in the Eµ-*TCL1* mouse model mirror human CLL and identify *Myc* as oncogenic hit associated with disease progression in patients.

## Introduction

Cellular heterogeneity and clonal evolution of tumors are of major interest in the era of targeted therapies, as these likely impact differently on specific cancer clones. During treatment, some tumor clones get reduced, but inadvertently drugs also provide potent selective pressure for expansion of other clones, a major cause of therapy resistance [1]. Chronic lymphocytic leukemia (CLL) is a malignancy of mature B cells in which genetic driver lesions and their relationship to clonal evolution have been identified [2], and an association of clonal evolution with treatment relapse and drug resistance has been described [3].

Eµ-*TCL1* mice are the most accepted and widely used mouse model of CLL for studying disease biology and for preclinical drug testing. This mouse model line was established by exogenous expression of the human *TCL1* gene under the control of the immunoglobulin heavy chain variable region (IGHV) promoter and IGH (Eµ) enhancer [4]. Starting from 6 months of age, these mice develop a CLL-like disease characterized by an accumulation of CD5^+^ B cells in blood and lymphoid organs, affecting almost 100% of the animals. Usually only at an age of more than 12 months, mirroring the median age of 70 for CLL diagnosis in humans [5], these mice develop end-stage disease with splenomegaly and very high leukemic cell counts in blood. The expanded leukemic cells exhibit clonal immunoglobulin rearrangements and B-cell receptor (BCR) sequences without IGHV hypermutations, but stereotyped heavy-chain complementarity-determining region 3 (HCDR3) regions as similarly observed in about 30% of patients with CLL [6]. Although the *TCL1* gene is variably expressed in CLL patients, higher expression is associated with unmutated IGHV status, a characteristic that correlates with more aggressive disease and shorter lymphocyte doubling time [7, 8]. Accordingly, the Eµ-*TCL1* mouse model is widely accepted as a model of aggressive CLL and has been extensively used to study the role of mouse orthologs of various genetic players in CLL such as *BTK*, *PRKCB*, and *TP53* [6, 9–11]. The long latency of leukemia development in this model suggests that *TCL1* overexpression acts as a predisposing factor or initial hit for premalignant transformation, yet other genetic or microenvironmental aberrations appear to be required for full leukemic transformation [12]. Consequently, this raises the possibility that the gain of genomic aberrations plays an active role in disease development in these mice. However, this has not been investigated intensively so far.

In this study, we aimed to identify the genomic aberrations, which might contribute to the leukemia development in the Eµ-*TCL1* mouse model, as well as study the clonal evolution using serial transplantation of *TCL1* leukemia. To achieve this, we performed whole exome sequencing (WES) as well as targeted sequencing of the complementarity-determining region 3 of the BCR locus of malignant B cells isolated from leukemic Eµ-*TCL1* mice and from animals after serial transplantation of leukemia cells in syngeneic wild-type (WT) mice. We observed that about half of the tumors were monoclonal, and oligoclonal tumors consisted of one major BCR clone. In addition, an increased mutational load, partly accompanied by BCR changes was observed upon transplantation of tumors. Most strikingly, all tumors but one showed a gain in chromosome 15, and therefore *Myc*, which we propose to be the main second hit for tumor formation in this mouse model.

## Results

**Fig. 1:**
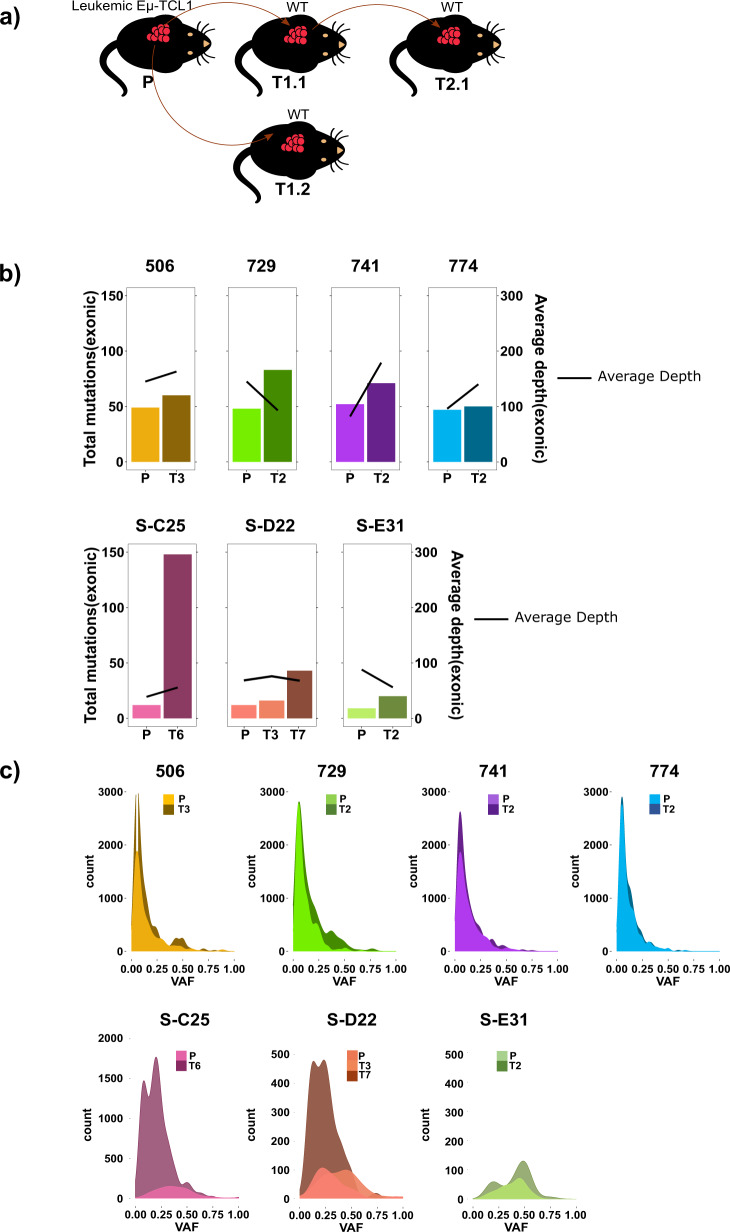
Exonic mutations in tumors of Eµ-TCL1 mice. **a** Scheme showing the transplantation of tumors isolated from spleens of primary Eµ-*TCL1* mice (P) to serially transplanted mice (T1, T2 etc). **b** Total exonic mutations as well as average sequencing depth at the targeted region is depicted for primary Eµ-*TCL1* tumors (P) and matched serially transplanted tumors (T3: 3× transplanted, T2: 2× transplanted, etc), in the upper panel for samples from this study and in the lower panel for samples from SRP150049 cohort (samples denoted by S-). **c** Variant allele frequency (VAF) distributions of mutations in primary Eµ-*TCL1* tumors and matched serially transplanted tumors are shown, in the upper panel for samples from this study and in the lower panel for samples from SRP150049 cohort.

To characterize the genetic complexity and evolution of malignant B cells in the Eµ-*TCL1* mouse model of CLL, we performed WES of purified splenic B cells and germline controls of eight Eµ-*TCL1* mice manifesting CLL-like symptoms (primary tumor), as well as four mice, which were serially transplanted with malignant B cells of four different primary tumors (Fig. 1a). Average sequencing coverage at targeted regions for eight tumor samples (primary vs. transplanted) from our cohort was ~180×, except for the four lowly sequenced primary samples in which the average coverage was 80× (Table S1). We further included a recently published dataset (SRP150049) of WES of *TCL1* tumors [13] in the analysis. The workflow utilized for this analysis is outlined in Fig. S1. We detected a mutation load in the range of 0.1–2.0 per Mb, which is in line with the low mutation rate detected in CLL patients, which is typically around 0.8 per Mb [14].

In both cohorts, serial transplantation of tumors resulted in an increase in mutational load (somatic nucleotide variants (SNVs) and small Indels/Mb in coding regions; Fig. S2). This observation was independent of the sequencing depth at the targeted regions (Fig. 1b). Variant allele frequency distributions of mutations in all primary and transplanted tumors of our study showed that most mutations have a low-allele frequency (5–10%; Fig. 1c, upper panel), which may imply existence of several clones and subclones that might evolve when subjected to selection pressure after transplantation. Although the total number of mutations are lower in the SRP150049 dataset, interestingly, allele frequencies were mostly higher than in samples of our study (Fig. 1c, lower panel). Although penetrance of leukemia development in Eµ-*TCL1* mice is near 100%, the course of disease is quite heterogeneous. Therefore, adoptive transfer (AT) of malignant B cells in syngeneic WT mice is frequently used to create cohorts of mice with a more homogenous disease course, which is especially important for preclinical drug testing. Even though most tumors engraft in mice when the genetic background of the recipient and the donor matches, still some tumors are rejected upon transplantation [15]. Furthermore, transfer of one primary *TCL1* tumor in several age- and sex-matched recipient mice results frequently in individual differences in tumor development, making analysis of treatment effects challenging in this model (unpublished observations and Table S2). We monitored disease development after transplantation of 14 different primary *TCL1* tumors into 4–14 recipient mice each by regular blood collection and analyses (Table S2). As we observed different degrees of heterogeneity in engraftment and leukemia development in the transplanted mice, we analyzed the BCR sequences of these tumors by RACE-PCR to elucidate their clonal composition in depth (Tables 1 and S3). Tumors with several subclones at a considerable percentage showed a heterogeneous engraftment pattern (e.g., Eµ-*TCL1* 647) or did not engraft at all (e.g., Eµ-*TCL1* 684) (Tables 1 and S2). Interestingly, some aggressive tumors growing in a very short time became monoclonal in the recipient mice by loss of previously existing minor subclones (e.g., Eµ-*TCL1* 1000; Tables 2 and S2).

**Table 1:** *Ighv* gene composition of 22 primary Eµ-*TCL1* tumors as revealed by targeted sequencing followed by RACE-PCR.

Recurrent variable genes are colored.

**Table 2:** *Ighv* gene composition of six primary Eµ-*TCL1* tumors and their subsequent transplantations as revealed by targeted sequencing followed by RACE-PCR.

Recurrent variable genes are colored. Primary tumors are listed first, followed by respective serially transplanted tumors (e.g., 774-2.1: tumor 774, transplanted 2 times, number 1 out of x).

**Fig. 2:**
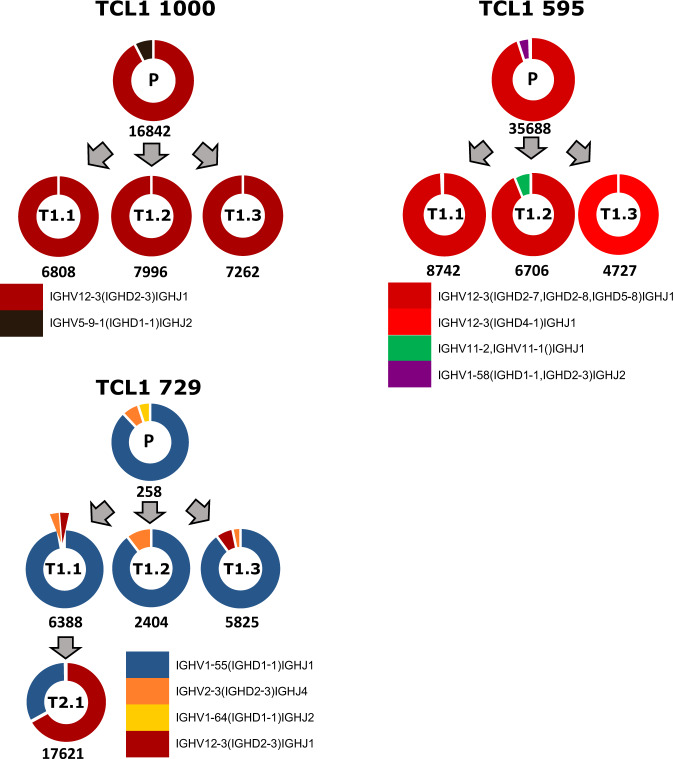
BCR clonotypes of Eµ-*TCL1* tumors. Eμ-*TCL1* BCR clonotypes as determined by RACE-PCR (all samples (*n* = 13) except 729 P) or exome sequencing (729 P) and identified using MiXCR. Each color represents a different V(D)J rearrangement, which is listed under each set of samples. Total number of reads in each sample is indicated below the donut plot and the sample number is indicated in the donut plot (P primary, T transfer).

In addition, BCR sequences from WES data of mouse tumors were used as input for MiXCR analyses for identifying tumor-specific V(D)J clonotypes. Six samples analyzed by both RACE-PCR and WES and resulting in the same V(D)J sequences, demonstrated that WES data can be also used to reliably predict V(D)J clonotypes (Table S3). However, as the read counts for the BCR genomic locus were low in WES, quantification was more reliable by RACE-PCR. Eight out of 22 sequenced primary tumors had monoclonal BCRs (≥99.5%). Seven had a dominant clone comprising more than 95% of the tumor. Only 4 out of 22 tumors comprised of several clones with a prevalence of more than 10% of the tumor. We detected stereotyped BCRs, which use the variable genes *Ighv1-55, Ighv11-2*, and *Ighv12-3* as the most frequent clonotypes of the malignant B cells (Table 1), which is in line with previous reports [6, 16]. Analysis of the BCR sequences of sequential tumor samples showed that the main BCR clone is conserved upon AT of tumors (Fig. 2 and Tables 2,  S3), except for 1 sample, where a clone with the same V and J chain, but with a new D chain emerged in one of the three recipients of the transplant (Fig. 2, TCL1 595). No reads were detected for the emerging new clone in the primary tumor (Table S3).

**Fig. 3:**
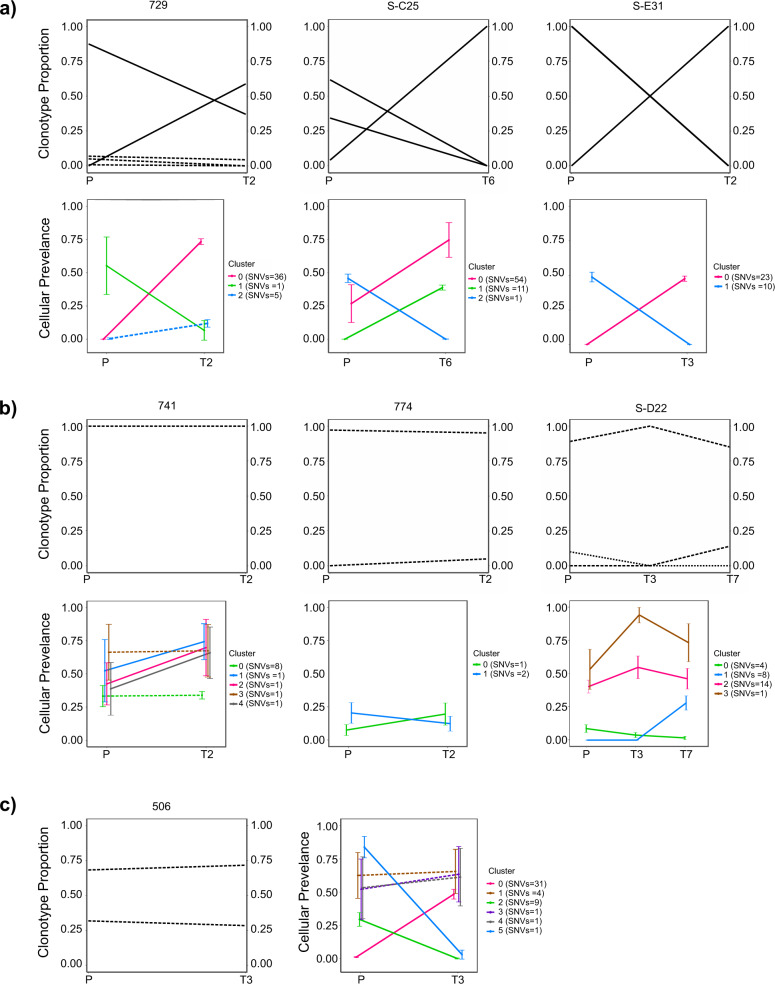
Clonal evolution of Eµ-*TCL1* tumors identified by changes in BCR clonotypes and as clusters of somatic variants showing similar allele frequency changes. Three types of clonal evolution patterns (**a–c**), attributed to changes in proportion of different BCR clonotypes (upper panels) and cellular prevalence of somatic mutation clusters (lower panels) between primary and serially transplanted tumors (P primary (*n* = 4), T transfer (*n* = 4)) are described. SNV cluster is a group of high confidence SNVs with similar allele frequencies at a time point. Changes from primary to transplanted tumors are denoted by solid lines. Changes occurring at <10% between primary and transplanted tumors are marked by a dotted line. Samples from SRP150049 (*n* = 6) cohort are denoted by “S-“ plus number of sample.

Next, we investigated the clonal evolution dynamics of these serially transplanted tumors with respect to somatic mutations as well as their BCR sequences. To identify evolving SNV clones, we used an approach to track dynamics of SNV clusters, i.e., groups of SNVs having similar cancer cell fractions at a time point rather than tracking clonal changes only by certain driver genes. For this, filtered SNVs (Table S4: in-house samples and Table S5: SRP150049 samples), their allele frequencies and copy number states as estimated using CNVkit, were used as input for PyClone that evaluated clusters of putative somatic clones and changes in the fractions of their cellular prevalence as the disease progressed in primary to transplanted tumors (Table S6) [17, 18]. Also, changes in proportions of BCR clonotypes from primary to transplanted tumors were noted. On tracking the dynamics of BCR clonotypes and changes in cellular prevalence of somatic mutations, several patterns of clonal evolution were evident. In the first pattern shown in Fig. 3a, tumors exhibited a change in the proportion of BCR clonotypes in the primary tumor vs. the transplanted tumor, and associated with that are changes in prevalence of SNV-defined subclones upon tumor transplantation. This pattern is indicative of an expanding new major clone that could be driven by acquired novel somatic SNVs in the transplanted tumors. In the second pattern, tumors showed stable clonotype proportions with B cells harboring one major BCR, and relatively stable SNV-defined subclones, indicative of a clonally stable disease before and after tumor transplantation without novel somatic SNVs arising (Fig. 3b). Interestingly, as a third pattern, one of the analyzed tumor pairs showed a stable BCR clonotype but major changes in SNV-defined subclones, indicative of a mutating tumor clone (Fig. 3c). Mutations associated with each of these patterns are marked in Tables S4 and S5. Overall, these results indicate that different clonal evolution patterns are observed in different tumors of Eµ-*TCL1* mice, which might depend on the varying proliferative capacities of the malignant cells and the presence of specific mutations providing advantages to some clones compared to others.

Furthermore, we examined mutations leading to amino acid changes that are predicted to affect protein function using SIFT analysis [19]. Using the criteria of an allele frequency of at least 10% and having a read coverage of the alternative allele of at least 5 and considering only the mutations changing across serial transplantations, we have detected deleterious mutations in the following 15 genes: *Speg, Ush2a, Obscn, Kcnk16, Pdia2, Grk2, Spi1, Ptpn3, Sema3a, Raf1, Smg1, Arhgef7, Ikbkb, AW551984*, and *Tbx18* (Table S4). Most of these mutations were detected only in the serially transplanted tumors, except for *Tbx18*, for which the allelic fraction of the mutation is 6.9% in the primary tumor and increases to 50.9% in the serially transplanted tumor. As *Tbx18* has been shown to have a role in proliferation and tumorigenesis, this mutation is likely to provide a growth advantage for the transplanted tumor [20]. Interestingly, we have observed a deleterious mutation for *Ush2a* in three out of four serially transplanted tumors. As Ush2a mutations have been shown to play a role in several leukemias and lymphomas, it is possible that this deleterious mutation contributes to the more aggressive phenotype of the transplanted tumors [21–23].

**Fig. 4:**
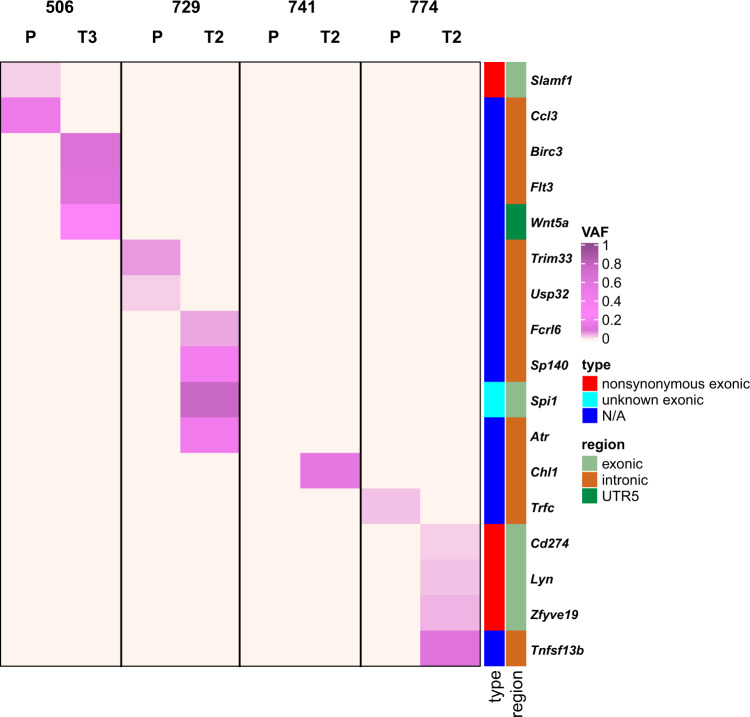
SNVs mapping to human CLL gene list from DISEASE database. Heatmap displaying variant allele frequencies (VAF) of 17 of the identified SNVs that map to the human CLL gene list (*n* = 309) from the DISEASE database at: https://diseases.jensenlab.org/. Types and regions of SNVs are displayed on the right of the heatmap. Samples of four paired tumors are depicted: P primary, T transfer, N/A not applicable.

We next investigated whether the detected mutations have pathogenic relevance in humans. We identified a total of 509 mutated genes across all our samples that were previously reported as being pathogenic in the COSMIC database v90 (Table S4), and there is a significant overlap with human pathogenic CLL genes in COSMIC (*p* < 1^−5^, Chi square test). Furthermore, we mapped the identified SNVs to the human CLL gene list (*n* = 309) from the DISEASE database (Fig. 4). Out of 17 genes that mapped to the DISEASE database, 7 have been linked to pathogenesis of human CLL and/or other leukemias (*Birc3, Wnt5a, SP140, Atr, Lyn, CD274*, and *Flt3*), and SIFT analysis revealed that the detected mutation in *Lyn* is likely deleterious (Table S4). These results suggest that disease development in the Eµ-TCL1 mouse model is driven by similar pathways as CLL in patients. For example, BIRC3 mutations (mostly nonsense and frameshift variants) were identified in fludarabine-refractory CLL patients, and were also used to define a high-risk CLL group [24]. Several kinds of dysregulation of the Wnt signaling pathway have been reported in leukemias [25]. Further, FLT3 mutations were found in one-third of newly diagnosed acute myeloid leukemia patients, and FLT3 internal tandem duplication was associated with relapse and inferior survival [26]. SP140 was one of the mutations identified in hyperdiploid multiple myeloma samples [27]. However, none of these mutations were recurring across our analyzed samples, and therefore attributed to clonal heterogeneity.

**Fig. 5:**
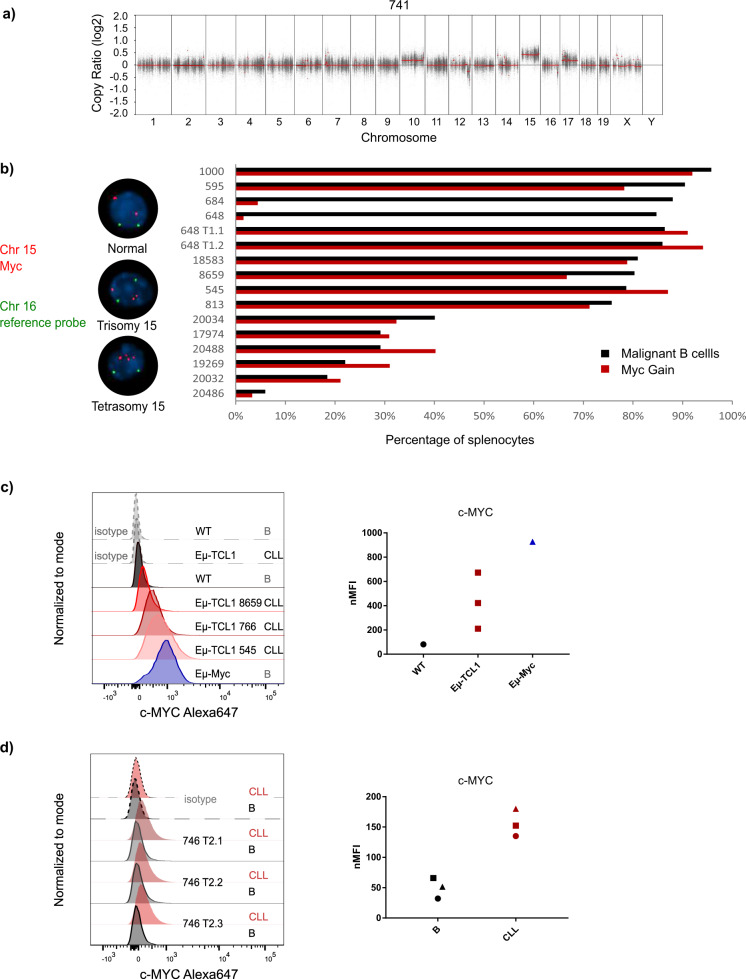
Gain of *Myc* in Eµ-*TCL1* tumors. **a** CNV plot of one exemplary sample (741) showing gain of Chromosome 15. **b** FISH analysis of splenocytes from 14 leukemic Eµ-*TCL1* mice for Chromosome 15 (*Myc* region) and Chromosome 16 as a control. Percentage of cells with gain of *Myc* and percentage of tumor cells identified as CD19^+^CD5^+^ are compared in the graph on the right. **c** Expression of c-MYC in CLL cells from three different leukemic Eµ-*TCL1* mice, as well as B cells from a WT mouse and an Eµ-*Myc* mouse, analyzed by flow cytometry. Dashed lines show the isotype control samples. **d** Expression of c-MYC in CLL cells (CD19^+^CD5^+^) and conventional B cells (CD19^+^CD5^−^) from three different leukemic mice after adoptive transfer, analyzed by flow cytometry. Dashed lines show the isotype control samples.

By analyzing the copy number variations in the tumor samples analyzed by WES (Table S7), we observed a deletion in chromosome 12 in almost all samples (Figs. 5a and S3), which is located within the immunoglobulin variable region, and therefore is expected to occur during VDJ recombination in B cells. Interestingly, in 13 out of 15 (87%) primary tumor samples (7/8 of our samples and 6/7 of SRP150049), a gain of chromosome 15 (chr15) was identified (Figs. 5a and S3). We further detected the same pattern of chr15 gain in the respective transplanted tumors (Fig. S3). We excluded that this was due to a chromosomal abnormality of the mouse line, as the germline samples did not show this gain. Interestingly, *Myc* oncogene is located on chr15 within the gained region in mice. To confirm the gain of *Myc* in malignant cells of Eµ-*TCL1* mice, we performed interphase FISH analysis of splenocytes from 14 leukemic Eµ-*TCL1* mice with primary disease, 3 non-leukemic, 5-week-old Eµ-*TCL1* mice, as well as 5 WT littermate mice from the Eµ-*TCL1* breeding (Fig. 5b and Table S8). In addition to a *Myc*-specific FISH probe, we used a chromosome 16 probe to exclude polyploidy of the tumor cells. Minor tetraploidy was detected in 10/17 *TCL1* samples (0.51% in 9 samples and 6.7% in 1 sample) and those cells were excluded from the analysis. Twelve out of 14 primary tumor samples showed trisomy of *Myc* in almost all tumor cells (Fig. 5b; exception #684 and #648 with <10% of cells with *Myc* gain). Interestingly, both mice with early or late stage (reflected by the tumor load) of the primary disease showed *Myc* gain (Fig. 5b and Table S8), suggesting that *Myc* gain is an early event in malignant transformation in this model. In 11 of these tumor samples, tetrasomy of the *Myc* locus was also detected, although at a much lower frequency (0.5–5% of analyzed cells) (Table S8). We have not detected more than four copies of *Myc* in any of the samples. By analyzing healthy WT and non-leukemic Eµ-*TCL1* mice, a very low frequency of trisomy 15 was detected in one out of three non-leukemic Eµ-*TCL1* samples (3% of cells) as well as two out of five WT samples (3% of cells) (Table S8). Remarkably, one tumor sample (#684) that showed *Myc* gain only in 4% of the cells (Fig. 5b) did not engraft in any of the transplanted mice (0 out of 10 mice; Table S2), suggesting that gain of *Myc* is necessary for tumor cell engraftment. Further, another tumor sample (#648) with a very low percentage of *Myc* gain (Fig. 5b) engrafted only in two out of four transplanted mice (Table S2), and in the engrafted tumors (#648 T1.1 and #648 T1.2), trisomy of *Myc*, however, was detected in all CLL cells (Fig. 5b; Table S8).

In order to test if the gain of the *Myc* locus also leads to enhanced expression of c-MYC protein, we performed intracellular flow cytometry. Normal B cells from WT mice did not express any detectable levels of c-MYC (Fig. 5c). On the other hand, CLL cells from Eµ-*TCL1* mice showed varying levels of c-MYC expression, with some mice expressing it at a similar level as B cells from Eµ-*Myc* mice (Fig. 5c), which harbor a B-cell-specific transgenic expression of *Myc* via the IGVH promoter and Eµ enhancer and develop a very aggressive lymphoma [28, 29]. To compare the c-MYC expression in normal B cells and CLL cells from the same mice, we used three mice adoptively transferred with Eµ-*TCL1* cells, where the normal B cells originate from the recipient WT mice. In these mice, we observed that c-MYC expression was specific to CLL cells and almost undetectable in normal B cells (Fig. 5d).

**Fig. 6:**
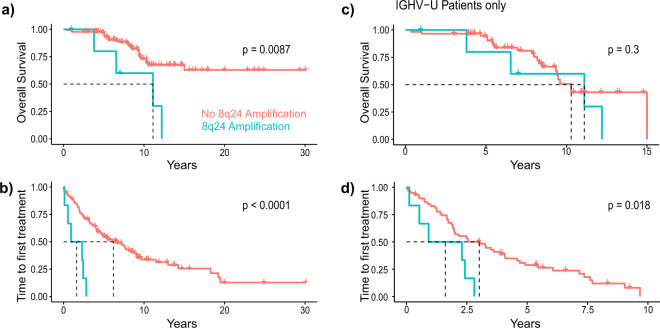
8q24 amplification in CLL patients. **a** Overall survival and **b** time to treatment analysis of CLL patients with (*n* = 6) or without (*n* = 128) 8q24 amplification (blue and red line, respectively). **c** Overall survival and **d** time to treatment analysis within patients with unmutated IGHV genes with (*n* = 5) or without (*n* = 60) 8q24 amplification.

Dysregulation of *MYC* is essential in the pathogenesis of a number of B-cell lymphomas [30]. Besides diffuse large B-cell lymphoma and Burkitt lymphoma, *MYC* expression has been linked to Richter’s syndrome, which is the transformation of CLL into a more aggressive B-cell lymphoma. In a cohort of 134 CLL patients, an amplification of chromosome 8q24, encompassing the *MYC* locus in humans, was detected in six samples. Although found rarely in CLL patients, this amplification caused a disadvantage in survival as well as shortened the time to next treatment compared to the patients without this amplification (Fig. 6a). As almost all patients with 8q24 amplification harbored an unmutated IGHV locus (5/6, for one patient unknown), which is associated with shorter survival, we also compared overall survival and time to first treatment only within the cases with unmutated IGHV. Although 8q24 amplification did not affect survival within this patient group, it still led to a worse outcome with respect to time to first treatment (Fig. 6b). These results suggest *MYC* as an oncogenic driver of progressive CLL also in patients.

## Discussion

CLL is a B-cell malignancy driven by antigenic stimulation of the BCR, as well as genetic aberrations affecting different cellular pathways, including RNA processing, DNA damage and cell cycle control, chromatin modifications, Notch and Wnt signaling, and inflammatory pathways [2]. Clonality analysis of WES data of CLL patients allowed the identification of temporal relationships between driver events, and a direct comparison between matched pretreatment and relapse samples demonstrated highly frequent clonal evolution in CLL [2]. So far, only limited data of genetic aberrations in the Eµ-*TCL1* mouse model of CLL and their temporal behavior during disease progression exist [13].

Similar to human CLL, nonoverlapping low-allele frequency SNVs (<5%) were identified in primary and serially transplanted tumor samples of the Eµ-*TCL1* mouse model using WES. Among them, we detected SNVs in genes that have been shown to be of relevance in the pathogenesis of human CLL and/or other leukemias. The most prominent one was *Birc3*, which belongs to the recurrently mutated genes in CLL patients and was found to be associated with increased risk of CLL progression [31, 32]. Another mutated gene, *Wnt5a*, is a member of the Wnt signaling pathway and was previously shown to bind to ROR1 and thereby to contribute to migration and proliferation of CLL cells [33, 34]. A genetic polymorphism in the gene *SP140* has been linked to an increased risk to develop CLL [35], making the identified *SP140* mutation of interest. Mutations in the genes *CD274* (coding for PD-L1) and *Lyn* are of interest, as the importance of these genes in CLL development was shown both in patients and in the Eµ-*TCL1* mouse model [36–39]. Other genes of interest are *Flt3* and *Atr*, which were shown to be upregulated and to have a pathological role in CLL and other leukemias [40–43]. Future follow-up work will be necessary to explore the relevance and underlying mechanism of these or other mutated genes for leukemia development in the Eµ-*TCL1* mouse model, and to estimate the usefulness of *TCL1* tumors harboring these mutations as preclinical models for CLL.

As 30% of CLL patients express quasi-identical BCRs, the so-called “stereotyped” receptors, the existence of common antigenic determinants as drivers of disease was suggested [44]. Both autoantigens arising upon apoptosis or oxidation-specific epitopes, and exogenous microbial antigens were identified as epitopes for these stereotyped BCRs. Of clinical importance, subsets of CLL patients with restricted BCRs have been identified and associated with clinical outcome [44]. Whereas stereotyped subsets #2 and #8 using the variable genes *IGHV3-21* and *IGHV4-39*, respectively, are linked to poor prognosis, patients of stereotyped subset #4 using the immunoglobulin genes *IGHV4-34* and *IGKV2-30* develop indolent disease [45]. In addition, CLL-specific HCDR3 regions of the BCR were shown to harbor antigen-independent cell-autonomous signaling, which is dependent on an internal epitope of the BCR [46]. The characteristics of BCRs expressed by leukemic B cells of the Eµ-*TCL1* mouse model are very similar to what is known in patients, with stereotyped BCRs reactive for microbial or autoantigens, the main one being phosphatidylcholine (PtC), identified as drivers of leukemia development [6]. Further, selection for PtC-reactive B cells was shown to increase the aggressiveness of the leukemia in the Eµ-*TCL1* mouse model [16]. This is in line with our findings that identified the PtC-reactive, stereotyped BCRs using the variable genes *Ighv11-2* and *Ighv12-3* as the most frequent clonotypes of the malignant B cells, together with *Ighv1-55*. Autoreactive CD5^+^ B cells with restricted BCRs were shown to be the origin of CLL development in aging mice [47], in which chronic stimulation by autoantigens causing persistent inflammation adds to leukemia progression. This confirms that CLL development in the Eµ-*TCL1* mouse model is driven by antigenic stimulation of the BCR.

Even though CLL is defined as a disease of monoclonal B cells having a unique *IGH* gene rearrangement, with increasing sequencing sensitivity a considerable fraction of cases with more than one clonotype was detected [48–50]. Two productive *IGH* rearrangements were discussed to arise in a single CLL cell, which might not follow the rule of allelic exclusion allowing only one productive *IGH* rearrangement. However, single-cell sequencing showed that allelic exclusion was generally maintained in CLL, and multiple productive *IGH* rearrangements are rather derived from distinct/unrelated clones in selected cases [48]. Bi- or multiclonality in CLL was shown to comprise of either a minor and a major, or equally sized clones, which persisted in patients over time and treatment. Even though the number of analyzed cases is small, multiclonality seemed to be more abundant in CLL cases with mutated *IGHV* genes, which is the group of patients with less aggressive disease. This is in line with our findings in the Eµ-*TCL1* mouse model, in which multiclonality is rare and associated with an inferior engraftment rate of leukemic cells in WT mice representing a feature of lower aggressiveness. A previous study compared clonality of malignant B cells in the Eµ-*TCL1* model at 4 and 8 months of age and detected several clonotypes at the earlier time point, whereas one major BCR rearrangement was observed in 8-month-old mice suggesting monoclonal expansion of CLL cells during progressive disease [51].

By combining patterns of BCR dynamics and associated SNV-defined subclones, we investigated tumor heterogeneity and evolution in serially transplanted tumors of the Eμ-*TCL1* mouse model. Three patterns of evolving SNV-defined subclones and BCR clonotypes emerged from primary to transplanted tumors. In the first pattern, both BCR clonotypes as well as somatic variants were displaced by novel clones after serial transplantation of the tumors. In the second pattern, the same BCR clonotypes and SNV-defined clones remained stable at the primary and transplanted time points. The third pattern showed tumors in which the BCR clonotypes remained constant but genetic changes were observed by shifts in SNV-defined clones. The variation in clonal evolution likely depends on the potency of the BCR clones as well as the accumulation of somatic variants and thereby affected genes. The dynamic loss and gain of BCR clonotypes as well as SNV-defined subclones could be indicative of differences in the selection pressure mediated by the tumor microenvironment, including the strength of antitumor immune responses during the course of CLL. Clonal evolution has been shown to be heterogeneous also in patients with CLL and mainly driven by treatment [2]. However, also in untreated CLL, dynamic changes in the disease course of CLL were shaped by the genetic events that were already present in the early slow-growing stages [52]. Especially, subclones with aberrations of known CLL driver genes were shown to harbor a growth advantage over other clones and to display accelerated growth.

Among the well-recognized cancer drivers, *MYC* appeared to be affected by several recurrent genetic aberrations in CLL[2]. MYC activity has been mainly associated with aggressive, high grade B-cell malignancies [30], and mice with overexpression of *Myc* in B cells (Eµ-*Myc*) develop an aggressive lymphoma/leukemia [53]. Although it is rare, an amplification of 8q24, the genomic locus of *Myc* in humans, has been associated with relapsed/refractory CLL cases previously treated with chemo(immuno)therapy [54]. Furthermore, 8q24/*MYC* gain is often acquired during the course of disease in CLL and not found in the early stages of the disease. If presented in a complex karyotype, it is frequently associated with Richter’s transformation, refractoriness to therapy and an aggressive clinical course [55]. This is in line with our data that clearly show a shorter overall survival of CLL patients harboring an 8q24 amplification. In the Eµ-*TCL1* mouse model, we observed a chromosomal gain of *Myc* in almost all analyzed tumors which resulted in an aberrant expression of MYC protein in the malignant B cells up to the level of B cells from the Eµ-*Myc* mouse model. Interestingly, lack of this gain was linked to a low engraftment rate of tumors upon transplantation. Altogether, our data suggest that *MYC* is a potent driver of CLL development, and the Eµ-*TCL1* mouse line as preclinical model for aggressive, *MYC*-driven CLL.

## Methods

A detailed description of methods and analyses can be found in a supplementary file.

### Mouse models

Eµ-*TCL1* (*TCL1*) mice on C57BL/6 background were kindly provided by Carlo M. Croce (The Ohio State University, Columbus, Ohio, USA) [4]. Characteristics of all Eµ-*TCL1* mice used for RACE-PCR and Exome-seq are described in Table S9. Characteristics of all Eµ-*TCL1* mice used for FISH are described in Table S8. Adoptive transfer (AT) of TCL1 tumors, collection of tissue samples from mice and flow cytometry were performed as previously described [56]. All animal experiments were carried out according to governmental and institutional guidelines and authorized by the local authorities (Regierungspräsidium Karlsruhe, Germany, permit numbers: DKFZ337, G-36/14, G-98/16, and G123/14).

### Mouse immunoglobulin repertoire sequencing

RNA was used for RACE-PCR according to previously described protocols [57, 58]. List of RACE-PCR oligonucleotides are provided in Table S9.

### Sequencing and alignment

Library preparation for targeted sequencing was performed using SureSelectXT Mouse All Exon kit from Agilent. The samples were subsequently sequenced on HiSeq2000 or HiSeq4000 platforms using 100-bp paired-end reads with 4 samples per lane according to the manufacturer’s instructions at the DKFZ Genomics and Proteomics Core Facility.

Data from WES and targeted sequencing can be viewed and downloaded at https://www.ebi.ac.uk/ena/browser/home under the project Id PRJEB42362.

### Patients and survival analysis

The study was approved by the Ethics Committee of the University of Heidelberg. For 136 CLL patients for which WGS or WES has been performed [59], time to first treatment and overall survival were calculated based on clinical follow-up.

## Supplementary information

Supplementary Methods

Suppl. Figure Legends

Suppl. Fig. 1

Suppl. Fig. 2

Suppl. Fig. 3

Suppl. Table 1

Suppl. Table 2

Suppl. Table 3

Suppl. Table 4

Suppl. Table 5

Suppl. Table 6

Suppl. Table 7

Suppl. Table 8

Suppl. Table 9
