# Supplementary material for: Longitudinal analyses of CLL in mice identify leukemia-related clonal changes including a Myc gain predicting poor outcome in patients
Source: Leukemia. 2021 Aug 20;36(2):464–75. doi: 10.1038/s41375-021-01381-4 (PMC8807396; doi:10.1038/s41375-021-01381-4)
Supplement: Supplementary file 1 — Supplementary Methods [file 41375_2021_1381_MOESM1_ESM.docx]

**Supplementary Methods**

**Mouse models**

Eµ-*TCL1* (*TCL1*) mice on C57BL/6 background were kindly provided by Carlo M. Croce (The Ohio State University, Columbus, Ohio, USA) and crossed at least 10 times to ensure C57BL/6J or C57BL/6N background (1). 6-10 weeks old female WT mice for adoptive transfer of *TCL1* tumor cells were purchased from Janvier Labs.

Characteristics of all Eµ-*TCL1* mice used for RACE-PCR and Exome-seq are described in Suppl. Table 8. Characteristics of all Eµ-*TCL1* mice used for FISH are described in Suppl. Table 7.

Adoptive transfer (AT) of TCL1 tumors was performed as previously described (2). Briefly, tumor cells were enriched from splenocytes of *TCL1* mice using EasySep™ Mouse Pan-B Cell Isolation Kit (Stemcell Technologies, 19844) according to the manufacturer’s protocol. The CD5^+^CD19^+^ content of purified cells was typically above 95 %, as measured by flow cytometry. 2x10^7^ of these enriched TCL1 splenocytes were transplanted by intraperitoneal (i.p.) injection into 6-10 weeks old C57BL/6 WT females.

All animal experiments were carried out according to governmental and institutional guidelines and authorized by the local authorities (Regierungspräsidium Karlsruhe, Germany, permit numbers: DKFZ337, G-36/14, and G-98/16, G123/14).

Sample size was determined based on expected variance of read-out. Samples or animals were excluded from the analyses if they did not develop CLL-like disease, either in the primary Eµ-TCL1 mouse line or after adoptive transfer of TCL1 leukemia cells. No randomization or blinding was used in animal studies.

**Collection of tissue samples and preparation of cell suspensions from mice**

Peripheral blood was drawn from the submandibular vein for measurements during ongoing tumor growth and by heart puncture at the endpoint of the experiment, and collected in ethylenediaminetetraacetic acid (EDTA)-coated tubes (Sarstedt). Mice were euthanized by increasing concentrations of carbon dioxide (CO_2_). Spleen single-cell suspensions were generated by using the gentleMACS tissue dissociator with Gentle MACS tubes C (Miltenyi Biotec) and passing the cells through 70 μm cell strainers (BD Biosciences). Erythrocytes were lysed by using Red blood cell lysis buffer (Biolegend, 420302).

**Flow cytometry**

After preparation of single-cell suspensions, cells were blocked for 15 min with rat serum, diluted 1:50 in PBS. Afterwards, the cells were incubated with recommended dilutions of antibodies (PerCP/Cyanine5.5 anti-mouse CD45, Biolegend 103132; FITC anti-mouse CD19, ThermoFisher Scientific 11-0193-82; PE anti-mouse CD5, Biolegend 100608) against cell surface proteins in PBS containing 0.1 % fixable viability dye (ThermoFisher Scientific, 65-0866-14) for 30 min at 4 °C. After washing twice with PBS/2 % FCS, cells were fixed using IC fixation buffer (Thermo Fisher Scientific, 00-8222-49), washed and stored in PBS/2 % FCS at 4 °C in the dark until analyzed by flow cytometry.

For intracellular c-Myc staining, cells were fixed after surface staining with Foxp3 fixation/permeabilization buffer (Thermo Fisher Scientific, 00-5523-00) for 30 min at RT, followed by permeabilization with 1X permeabilization buffer (Thermo Fisher Scientific, 00-5523-00) and then staining with an antibody against c-Myc (Alexa Fluor 647 D84C12) or an isotype antibody (Rabbit (DA1E) Isotype Alexa Fluor 647 #2985) in 1X permeabilization buffer for 30 min at 4 °C. After washing twice with 1X permeabilization buffer, cells were resuspended in 1X permeabilization buffer and stored at 4 °C in the dark until analyzed by flow cytometry.

For labelling of cells in whole blood, 25 µL of PB was stained with antibodies specific for surface molecules (PerCP/Cyanine5.5 anti-mouse CD45, Biolegend 103132; FITC anti-mouse CD19, ThermoFisher Scientific 11-0193-82; PE anti-mouse CD5, Biolegend 100608) for 30 min at 4 °C, followed by incubation for 10 min with 2 mL of 1-step Fix/Lyse Solution (Thermo Fisher Scientific, 00-5333-57) to remove erythrocytes. After centrifugation, supernatants were carefully aspirated, and pelleted cells were resuspended in PBS.

Flow cytometry data was acquired using a BD FACS Canto II or BD LSR Fortessa (BD Biosciences, Heidelberg, Germany) FACS analyzer and analyzed by FlowJo X 10.0.7 software (FlowJo, Ashland, OR, USA).

**Mouse immunoglobulin repertoire sequencing**

RNA quality was assessed with the Agilent Bioanalyzer and 300-500ng were used for RACE PCR according to previously described protocols (3, 4) with minor modifications. Briefly, cDNA was synthesized using primers annealing to heavy or light chain constant regions and a barcoded template-switching primer followed by AMPure bead purification. Two consecutive exponential PCRs were subsequently performed using 2 μl of the single-stranded cDNA or the first amplification product, respectively. Libraries were purified with AMPure beads and pools size selection was performed on agarose gels prior to 400+100bp paired-end sequencing in the Illumina MiSeq platform.

List of RACE PCR oligonucleotides are provided in Supplementary Table 9.

**Sequencing and alignment**

Library preparation for targeted sequencing was performed using SureSelectXT Mouse All Exon kit from Agilent. The samples were subsequently sequenced on HiSeq2000 (n=8, 4 Eµ-*TCL1* mouse tumors and matched controls); and HiSeq 4000 (n=12; 4 primary tumors, 4 secondary transplanted tumors, 4 matched controls from the primary tumors) platforms using 100bp paired-end reads with 4 samples per lane according to the manufacturer’s instructions at the DKFZ Genomics and Proteomics Core Facility.

Raw exonic reads (FASTQ) were aligned using Burrows-Wheeler Aligner (bwa-mem v0.7.8) to the mouse reference assembly (UCSC mm10) using default parameters. Biobambam2 (v0.0.148) was used for sorting. Duplicates were marked using Picard and temporary alignment files were merged.

Data from WES and targeted sequencing can be viewed and downloaded at <https://www.ebi.ac.uk/ena/browser/home> under the project Id PRJEB42362.

**Calling single nucleotide variants (SNVs)**

SNVs were called from aligned whole exome sequencing (WES) data of mouse tumors and matched control samples using the command line tool Mutect2 from GATK (v4.0.2.0) (5). The cohort included four primary Eμ-*TCL1* mouse tumors and their four serially transplanted secondary tumors. Additionally, all available normal/control mouse samples (n = 8; 3 primary tumor matched T cells from spleen and 5 primary tumor matched tails including 4 tails from separate Eμ-*TCL1* mice as additional controls) were used to construct a PON (panel of normals). Mutations were called first against each normal sample as if it was a tumor sample (tumor-only mode). Resulting germline mutations from all normal/controls were then combined using “CreateSomaticPanelOfNormals” functionality of Mutect2 and supplied during paired Mutect2 mutation calling. Other parameters used with Mutect2 were base quality score threshold of 25 (--base-quality-score-threshold) and dbSNP v142 for mm10 as a germline resource (--germline-resource) that helped to eliminate germline variants common in the population in general that were missed during mutation calling. Finally, pileup summaries (GetPileupSummaries) were estimated for each tumor bam file. Pileup summaries infer read support for a set number of known variants and this was used to calculate fraction of probable contaminants (CalculateContamination) in each sample. These contaminants were then filtered out from the Mutect2 paired tumor-matched normal mutation calls to generate a final variant call format (.vcf) file. Variants were then annotated with ANNOVAR (v2017Jun1) and all the downstream analysis was performed with variants annotated by Mutect2 as ‘PASS’ and by ANNOVAR as ‘somatic’ (6).

Mutations were also called and annotated for Eμ-*TCL1* tumor samples from publicly available dataset processed at Salzburg (SRP150049) following the procedure described above but with additional controls from Salzburg (n=7) used to prepare the PON. Mutations called from Mutect2 for each sample are documented in Supplementary Tables 4 and 5.

**Calling copy number variations (CNVs)**

The command line tool CNVkit (v0.9.7.dev0) with the default hybrid capture mode was used for detecting copy number variants from WES data of Eμ-*TCL1* mouse tumors (7). The reference used while calling was built with all Eµ-*TCL1* mouse control samples (n=8) and a target .gtf file (downloaded from Agilent for SureSelectXT mouse all exon kit) was supplied to infer CNV calls only from the genomic regions covered during sequencing. CNVs were similarly called from WES data of publicly available Eμ-*TCL1* mouse tumors (SRP150049). Focal CNVs across all samples are documented in Supplementary Table 6.

**Identification of B cell receptor rearrangements from WES**

B cell receptor rearrangements were quantified to identify V(D)J clonotypes from raw WES Eμ-*TCL1* mouse tumor data using MiXCR (v3.0.8)(8). The command line tool MiXCR mapped and assembled V, D and J gene segments from immunoglobulin region to report clonotypes for each tumor sample. The tool was run with default parameters: starting material - ‘DNA’, adapters - ‘present’ and receptor type - ‘IGH’. The position of V and J primers was mentioned to be 5’ and 3’ end respectively. The output file reported the frequency, number of supporting reads, CDR3 (complementarity determining region 3) amino acid sequence, CDR3 nucleotide sequence, the detected V, D and J genes for each assembled clonotype. If a clonotype was supported by more than 1% of total reads it was considered a true hit. All identified rearrangements are summarized in Supplementary Table 3.

**Identification of B cell receptor rearrangements after RACE-PCR**

BCR samples processed by RACE-PCR were analyzed by using MiGEC (9) and MiXCR (8) tools. MiGEC was used for the initial pre-processing of data for unique molecular identifiers (UMI) followed by the analysis of clones with MiXCR, as described above for WES samples.

**Calculation of change in cellular prevalence of mutations from primary to serially transplanted tumors**

To infer if identified SNVs were contributing to clonal changes in mouse tumors, allele frequency changes of tumor specific and shared (between primary and adoptive transfer tumor pairs) mutations were calculated. Python tool PyClone (v0.13.0) was used for calculating and plotting clonal changes between tumor pairs (10). PyClone used as input SNVs and copy number state information to output putative clonal population clusters corrected for allelic imbalances in each sample defining clonal shifts from primary to secondary tumor transfer. SNVs used for this analysis were filtered for most reliable hits as follows. VAF (variant allele frequency) cutoff of minimum 10% and depth of at least five reads support at any one time point (primary: time point 1 or transfer: time point 2) for each SNV identified using Mutect2 was considered. For SNVs present at only one time point, mpileup summaries were used to calculate the probability of existence of the SNV with at least two reads at the time point given the coverage at that position. Also, the SNV should have been called at the other time point with 10% VAF and at least five reads. To strictly avoid any technically artifactual SNVs, 1% error was allowed considering a 99% confidence interval for each SNV position. High confidence SNVs from here that passed adjusted p-value (Bonferroni correction (11)) threshold of <0.05 were selected and used for plotting clonal changes between tumor pairs.

**SIFT analysis**

The impact of the identified SNVs was assessed based on SIFT (12) using Ensembl's VEP (Variant Effect Predictor) version 102 with default settings in the VM provided by Ensembl.

**Fluorescence in situ hybridization (FISH)**

Two-color FISH experiments were performed using a BAC clone for the MYC gene (RP23-397P6) localized on mouse chromosome 15 and a reference BAC clone for mouse chromosome 16 (E4290Q) as described by Geigl *et al*. 2006 (13). Interphase-FISH results were validated using an automated scanning system (Applied Spectral Imaging, Edingen-Neckarhausen, Germany).

**Patients and survival analysis**

The study was approved by the Ethics Committee of the University of Heidelberg. Informed consent was obtained in advance. The study is compliant with all of the relevant ethical regulations regarding research involving human participants.

For 136 CLL patients for which WGS or WES has been performed (14) we calculated time to first treatment and overall survival based on clinical follow-up. Analysis has been performed using R (version 4.0.3), and the R packages survival (version 3.2-7) and survminer (version 0.4.8). Characteristics of patients are provided in Supplementary Table 10.

**References**

1. Bichi R, Shinton SA, Martin ES, Koval A, Calin GA, Cesari R, et al. Human chronic lymphocytic leukemia modeled in mouse by targeted TCL1 expression. *Proc Natl Acad Sci U S A.* 2002;99(10):6955-60.

2. Sadik A, Somarribas Patterson LF, Ozturk S, Mohapatra SR, Panitz V, Secker PF, et al. IL4I1 Is a Metabolic Immune Checkpoint that Activates the AHR and Promotes Tumor Progression. *Cell.* 2020.

3. Afzal S, Gil-Farina I, Gabriel R, Ahmad S, von Kalle C, Schmidt M, et al. Systematic comparative study of computational methods for T-cell receptor sequencing data analysis. *Brief Bioinform.* 2019;20(1):222-34.

4. Turchaninova MA, Davydov A, Britanova OV, Shugay M, Bikos V, Egorov ES, et al. High-quality full-length immunoglobulin profiling with unique molecular barcoding. *Nat Protoc.* 2016;11(9):1599-616.

5. Benjamin D, Sato T, Cibulskis K, Getz G, Stewart C, and Lichtenstein L. Calling Somatic SNVs and Indels with Mutect2. 2019.

6. Wang K, Li M, and Hakonarson H. ANNOVAR: functional annotation of genetic variants from high-throughput sequencing data. *Nucleic Acids Res.* 2010;38(16):e164.

7. Talevich E, Shain AH, Botton T, and Bastian BC. CNVkit: Genome-Wide Copy Number Detection and Visualization from Targeted DNA Sequencing. *PLoS Comput Biol.* 2016;12(4):e1004873.

8. Bolotin DA, Poslavsky S, Mitrophanov I, Shugay M, Mamedov IZ, Putintseva EV, et al. MiXCR: software for comprehensive adaptive immunity profiling. *Nature Methods.* 2015;12(5):380-1.

9. Turchaninova MA, Davydov A, Britanova OV, Shugay M, Bikos V, Egorov ES, et al. High-quality full-length immunoglobulin profiling with unique molecular barcoding. *Nat Protoc.* 2016;11(9):1599-616.

10. Roth A, Khattra J, Yap D, Wan A, Laks E, Biele J, et al. PyClone: statistical inference of clonal population structure in cancer. *Nat Methods.* 2014;11(4):396-8.

11. Armstrong RA. When to Use the Bonferroni Correction. *Ophthalmic Physiol Opt.* 2014.

12. Ng PC, and Henikoff S. SIFT: Predicting amino acid changes that affect protein function. *Nucleic Acids Res.* 2003;31(13):3812-4.

13. Geigl JB, Uhrig S, and Speicher MR. Multiplex-fluorescence in situ hybridization for chromosome karyotyping. *Nat Protoc.* 2006;1(3):1172-84.

14. Dietrich S, Oles M, Lu J, Sellner L, Anders S, Velten B, et al. Drug-perturbation-based stratification of blood cancer. *J Clin Invest.* 2018;128(1):427-45.
