# Supplementary material for: Longitudinal analyses of CLL in mice identify leukemia-related clonal changes including a Myc gain predicting poor outcome in patients
Source: Leukemia. 2021 Aug 20;36(2):464–75. doi: 10.1038/s41375-021-01381-4 (PMC8807396; doi:10.1038/s41375-021-01381-4)
Supplement: Supplementary file 2 — Suppl. Figure Legends [file 41375_2021_1381_MOESM2_ESM.docx]

**Supplementary Figure Legends**

**S1:** Workflow of analysis applied throughout this study.

**S2:** Mutational load of WES samples in primary and matched transplanted tumors. Samples analyzed in this study are depicted in red (n=8), and samples re-analyzed from SRP150049 cohort (Zaborsky *et al*) are in black (n=6). Each shape represents one sample. *p<0.05, Wilcoxon matched-pairs signed rank test.

**S3:** CNV plots of all samples analyzed by WES. **a)** High coverage samples (n=8) **b)** Low coverage samples (n=4) **c)** SRP150049 primary samples (n=4) **d)** SRP150049 primary and matched transferred samples (n=7).
