# Supplementary figures and images for: Longitudinal analyses of CLL in mice identify leukemia-related clonal changes including a Myc gain predicting poor outcome in patients
Source: Leukemia. 2021 Aug 20;36(2):464–75. doi: 10.1038/s41375-021-01381-4 (PMC8807396; doi:10.1038/s41375-021-01381-4)

Supp. Fig. 1

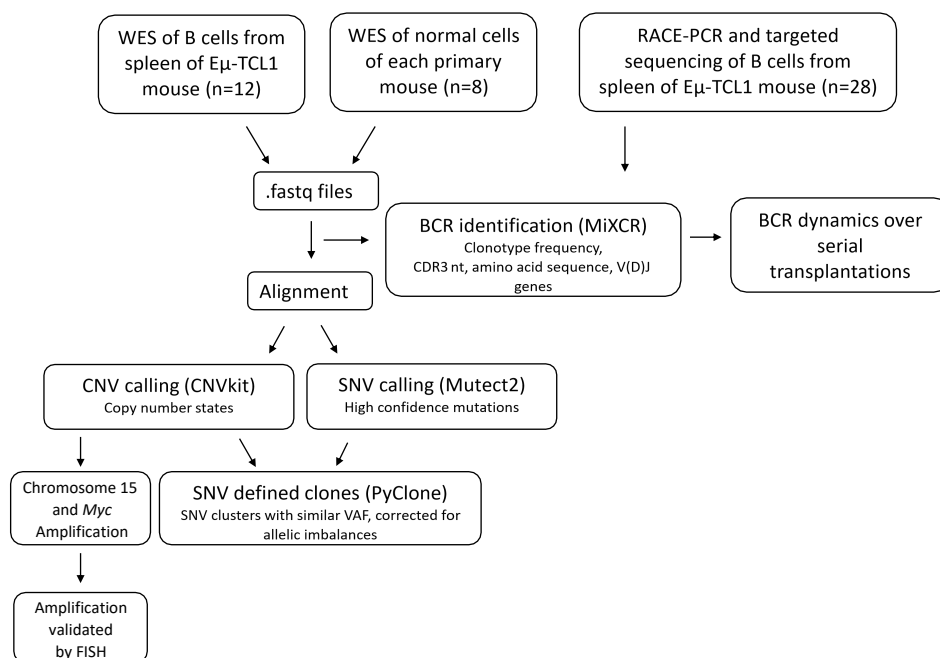

Supplement: Supplementary file 3 — Suppl. Fig. 1 [file 41375_2021_1381_MOESM3_ESM.pdf]

Sup. Fig. 2

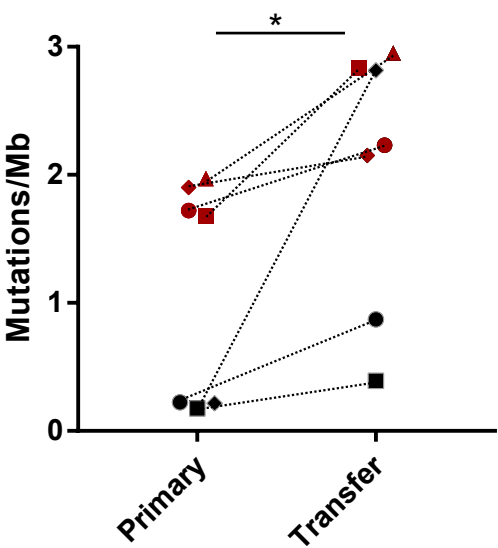

Supplement: Supplementary file 4 — Suppl. Fig. 2 [file 41375_2021_1381_MOESM4_ESM.pdf]
