# Supplementary material for: Longitudinal analyses of CLL in mice identify leukemia-related clonal changes including a Myc gain predicting poor outcome in patients
Source: Leukemia. 2021 Aug 20;36(2):464–75. doi: 10.1038/s41375-021-01381-4 (PMC8807396; doi:10.1038/s41375-021-01381-4)

Supp. Fig. 3

High coverage samples

a)

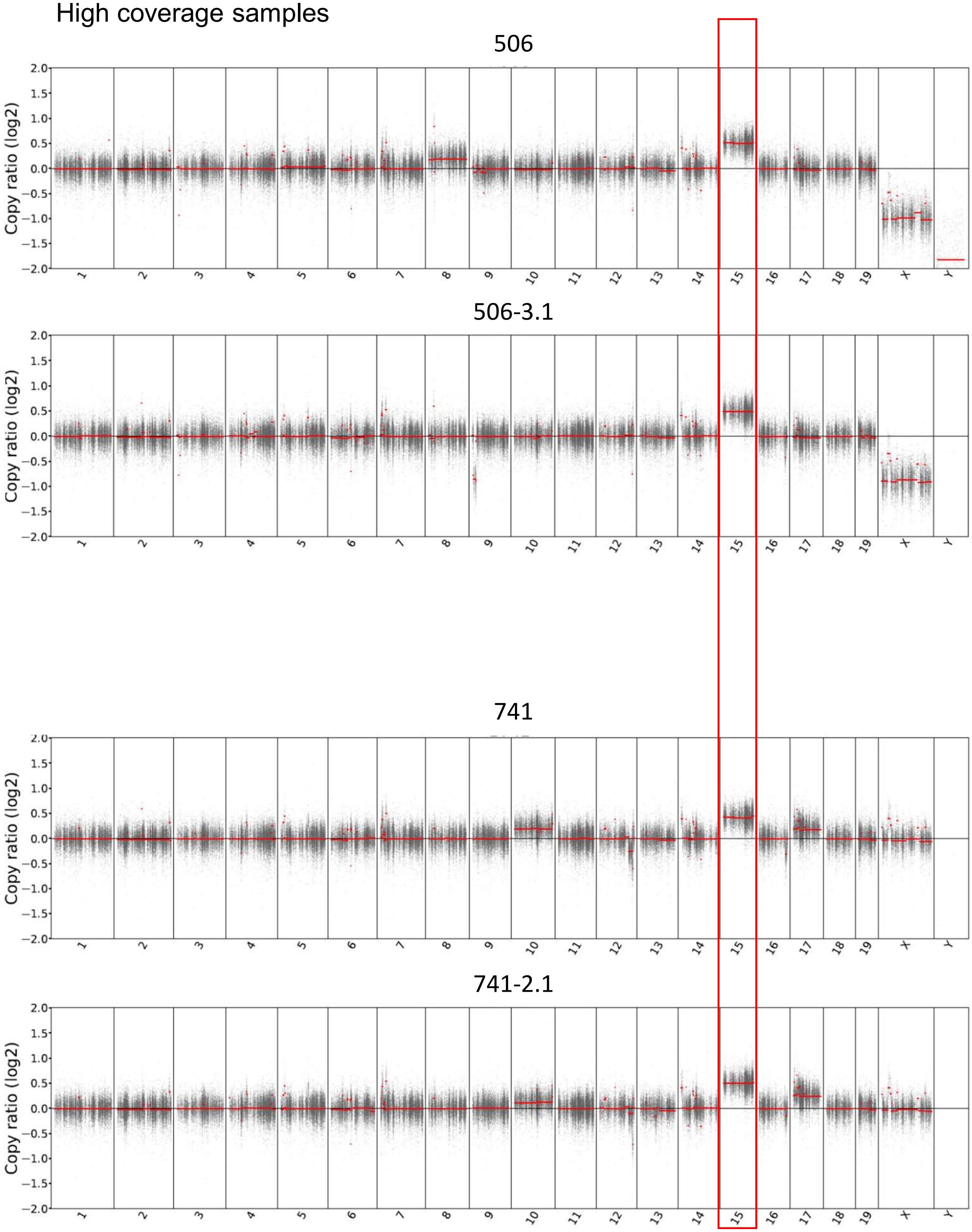

Supp. Fig. 3

High coverage samples

a) (continued)

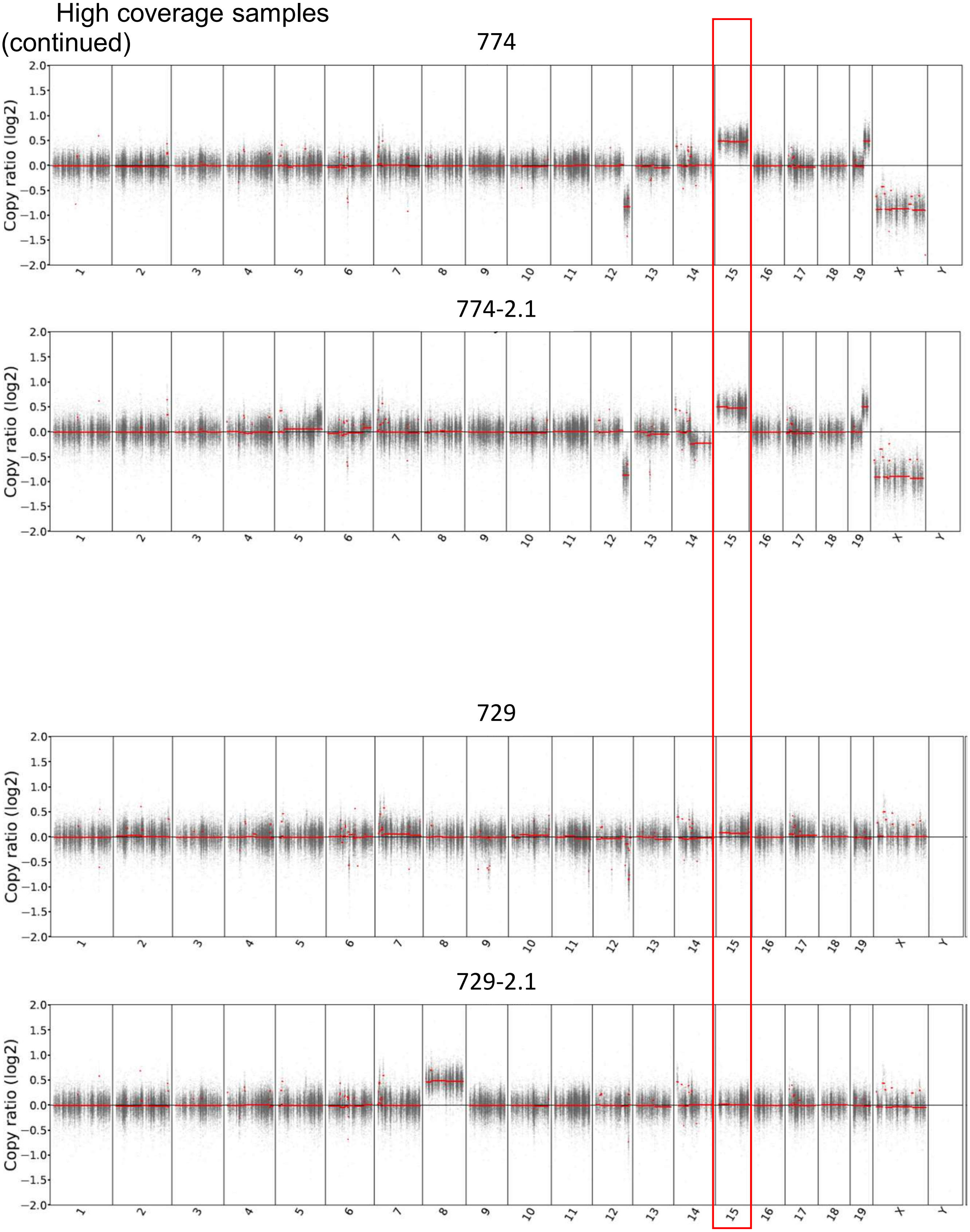

Supp. Fig. 3

Low coverage samples

b)

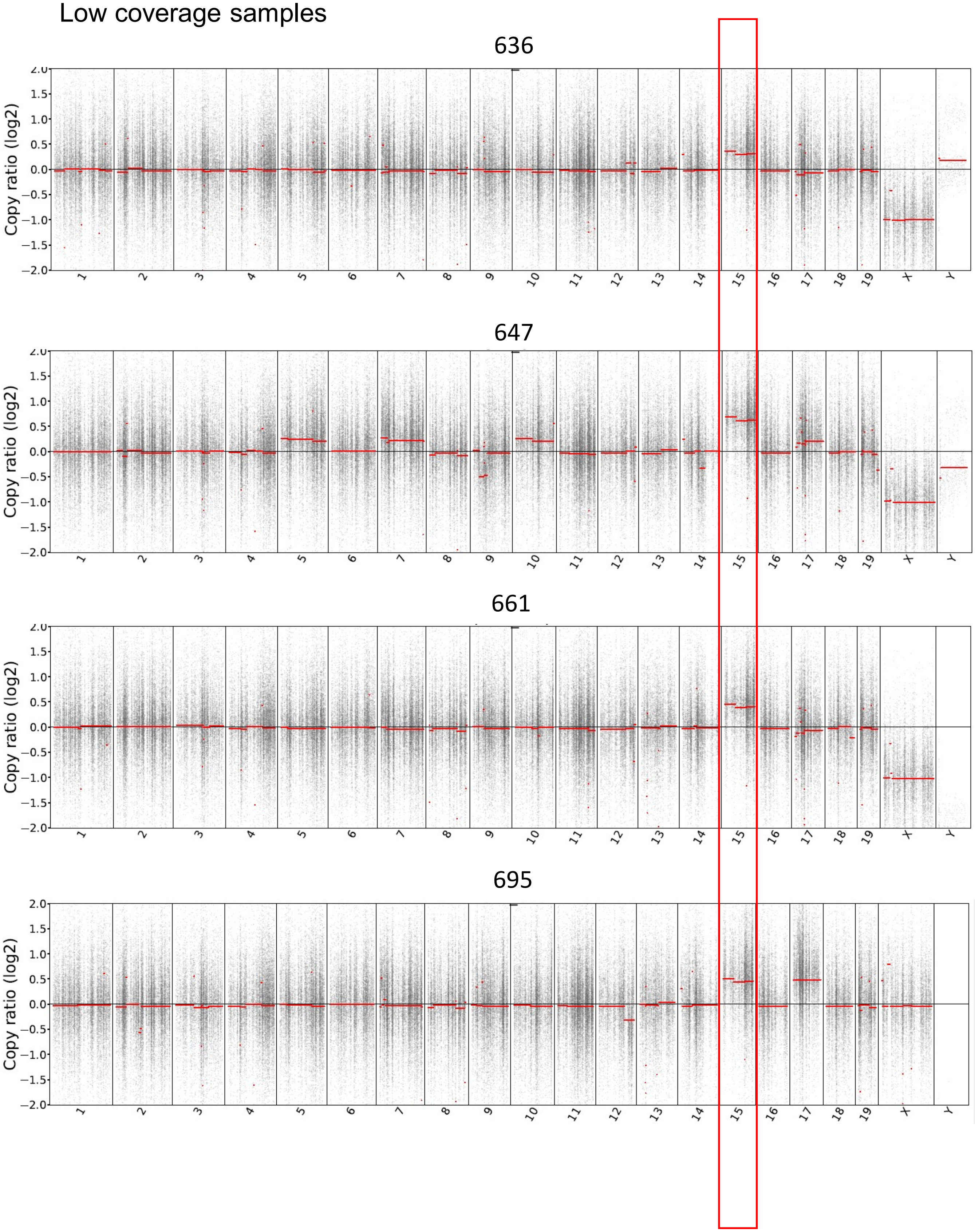

Supp. Fig. 3

SRP150049 primary samples

c)

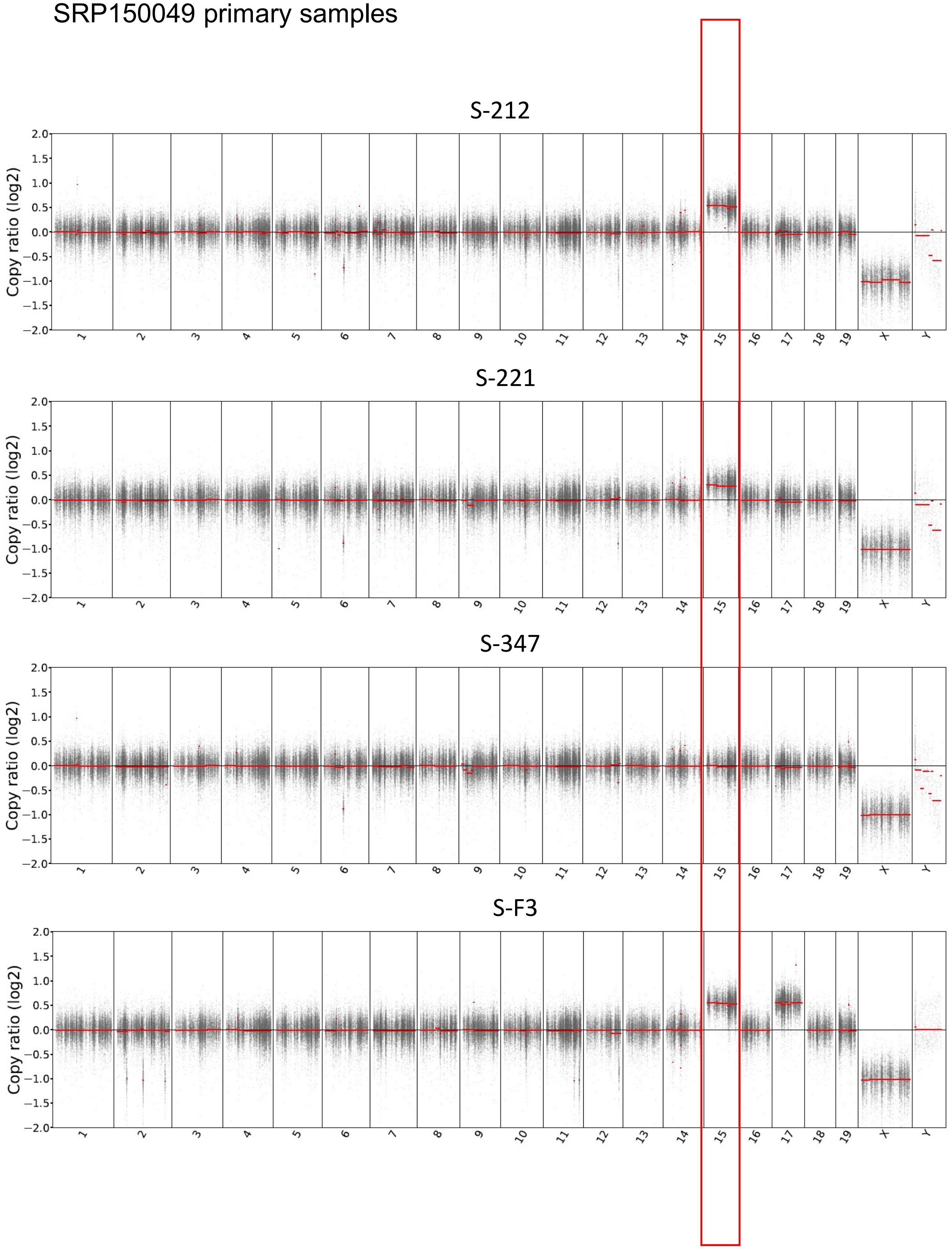

Supp. Fig. 3

SRP150049 primary and transferred samples

d)

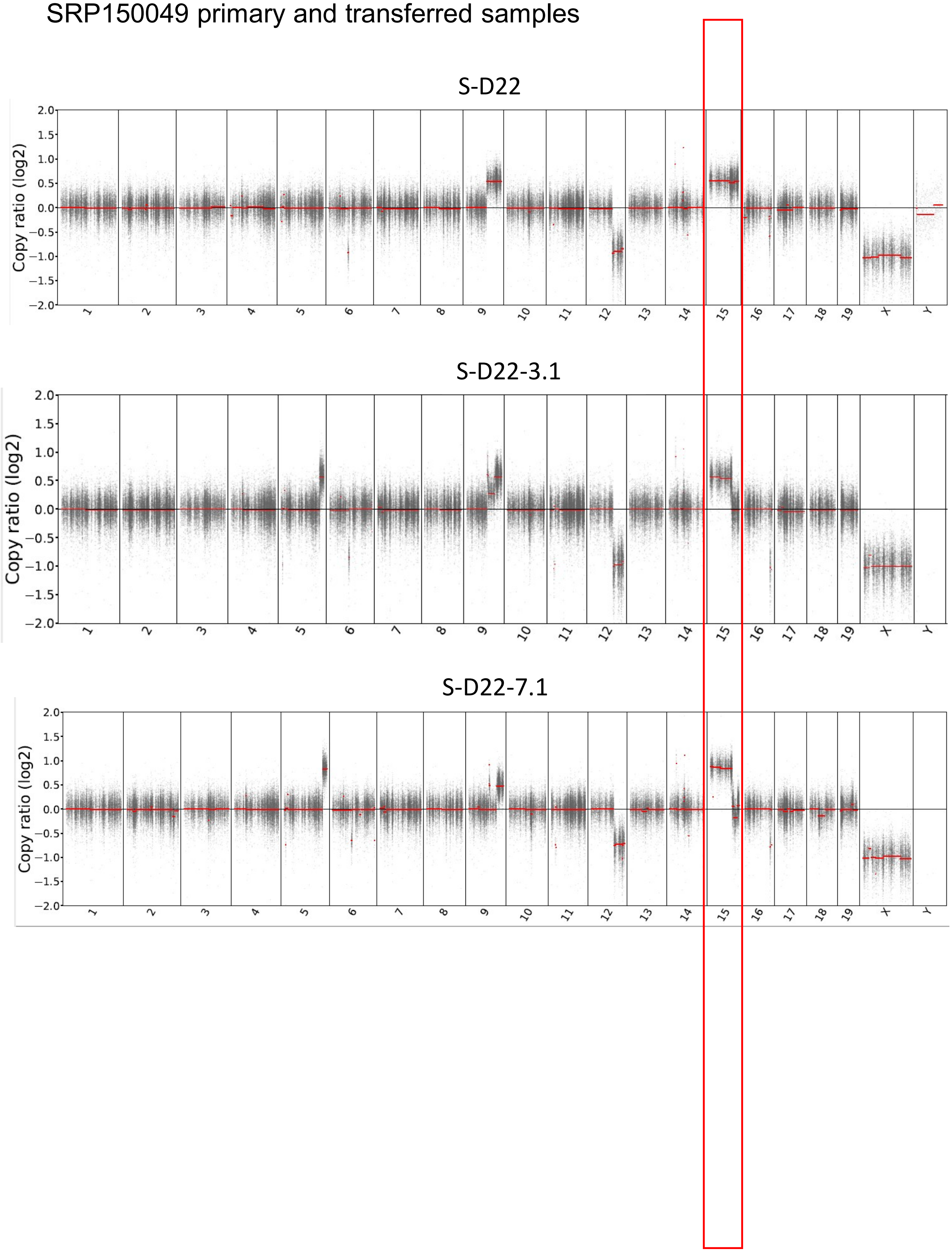

Supp. Fig. 3

SRP150049 primary and transferred samples

d) (continued)

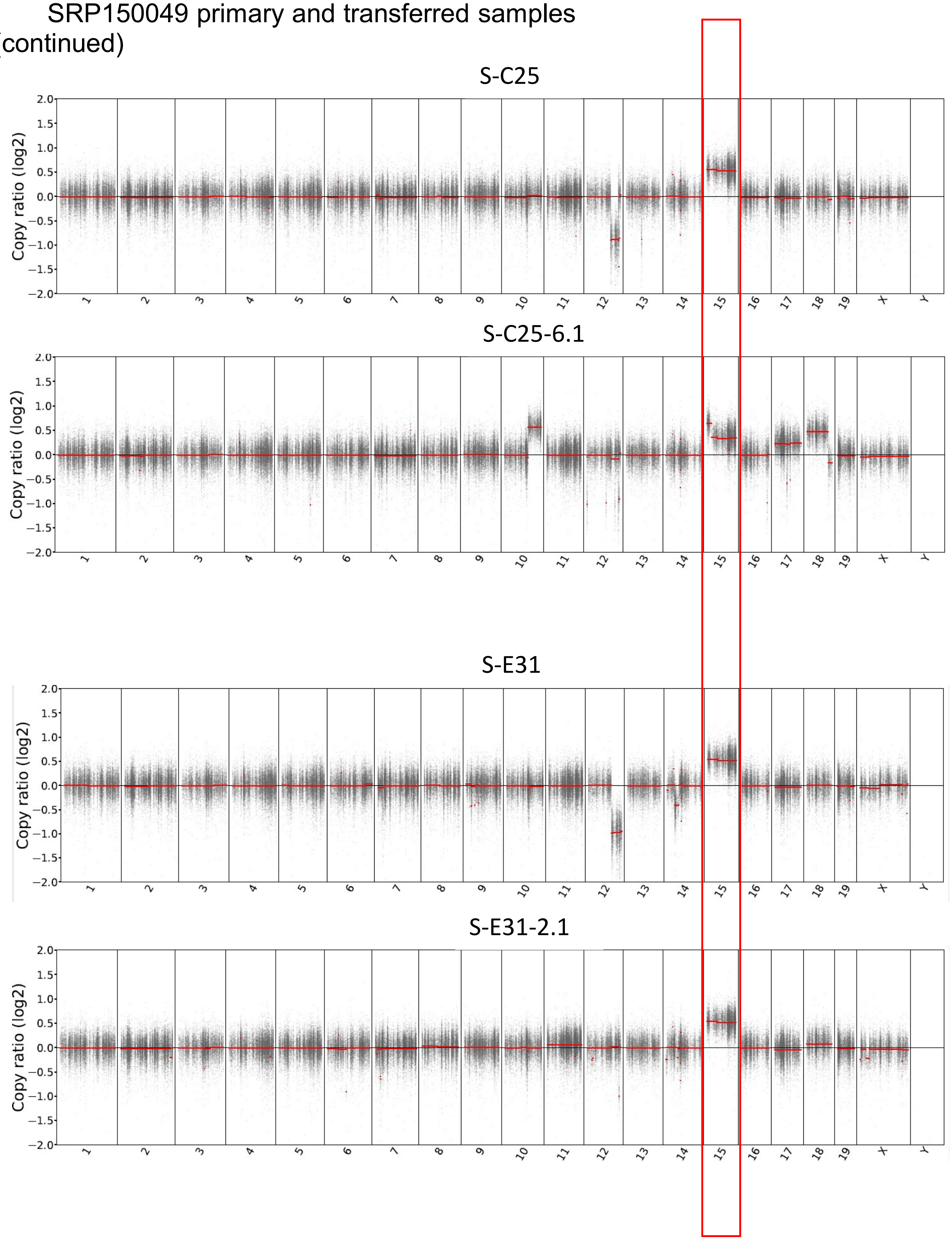

Supplement: Supplementary file 5 — Suppl. Fig. 3 [file 41375_2021_1381_MOESM5_ESM.pdf]
